# Cobalamin C Deficiency Presenting with CVID-like Immunologic Abnormalities: A Five-Patient Pediatric Case Series

**DOI:** 10.1007/s10875-026-02068-0

**Published:** 2026-08-24

**Authors:** Ruxuan He, Jinrong Liu, Shunying Zhao, Haiming Yang

**Affiliations:** Department of Respiratory Center, Beijing Children’s Hospital, Capital Medical University, National Center for Children’s Health, China National Clinical Research Center of Respiratory Disease, No. 56, Nanlishi Road, Beijing, 100045 P.R. China

**Keywords:** CblC Deficiency, CVID-like Immunologic Abnormalities

*To the Editor:* Cobalamin C (cblC) deficiency is the most common disorder of intracellular cobalamin metabolism and results from impaired synthesis of adenosylcobalamin and methylcobalamin, leading to combined methylmalonic acidemia and homocystinuria [1, 2]. Classic pediatric manifestations include poor feeding, failure to thrive, developmental delay, hypotonia, seizures or encephalopathy, cytopenias or megaloblastic anemia, ocular involvement, hemolytic uremic syndrome, pulmonary hypertension, and diffuse lung disease. Recurrent infections may occur but are not usually the dominant presenting feature. Beyond its well-characterized metabolic derangements, increasing evidence suggests that cblC deficiency may also impair immune function, leading to recurrent infections, hypogammaglobulinemia, and lymphocyte subset abnormalities that can mimic an inborn error of immunity (IEI). However, reports characterizing the immune status of patients with cblC remain limited. Here, we report five pediatric cases of genetically confirmed cblC deficiency presenting with CVID-like immunologic features. We detail their clinical, immunologic, and metabolic features to highlight this underrecognized overlap, emphasizing the importance of considering inborn errors of metabolism in the differential diagnosis of suspected primary immunodeficiency.

All five patients (four females, one male) presented with recurrent infections as the initial and predominant clinical manifestation. The median age at symptom onset was 2.3 years (range: 3 months to 5 years), with late-onset disease (> 1 year of age) observed in four patients. The most common presentations included recurrent respiratory tract infections, with pneumonia observed in four patients and upper respiratory tract infections in one. Additional infectious complications included viral encephalitis (*n* = 1) and *Candida albicans* infections (*n* = 2). Notably, there were no episodes of recurrent skin infections, *Pneumocystis jirovecii* pneumonia, or herpesvirus-related disease. Biochemical analyses confirmed the diagnosis of methylmalonic acidemia (MMA) in all cases according to European consensus guidelines [3]. Whole-exome sequencing identified compound heterozygous pathogenic variants in *MMACHC* in all patients. None of the patients had known relatives with recurrent or severe infections, diagnosed primary immunodeficiency, autoimmune disease, or known cblC deficiency. Autoantibody screening was negative in all cases. Comprehensive genetic evaluation did not reveal any mutations associated with known primary immunodeficiency syndromes.

Immunologic evaluation showed abnormalities resembling a primary antibody deficiency or CVID-like presentation, but these patients did not meet strict criteria for definite CVID (Table 1). Three of the five patients were younger than 4 years at the time of initial immunologic evaluation, vaccine responses were not systematically assessed, and persistent hypogammaglobulinemia was not documented in all cases. Humoral immune abnormalities were observed in four patients, with reduced IgG levels in three and low IgA and/or IgM levels in two patients. However, these findings alone do not establish intrinsic B-cell dysfunction. The normal IgG concentration in Patient 3 at 3 months of age may have partly reflected transplacentally acquired maternal IgG. Abnormal lymphocyte subsets were also noted, including CD3 + T-cell lymphopenia (*n* = 3), reduced CD19 + B-cell counts (*n* = 3), low NK-cell percentages in all five patients, and reduced absolute NK-cell counts in four of five patients. CD4/CD8 ratios were abnormal in two patients. Complete blood counts revealed leukopenia in three patients, lymphopenia in three, and neutropenia (absolute neutrophil count < 1500/µL) in three cases. These findings further indicate a pattern of global immune suppression associated with the underlying metabolic disorder.

**Table 1:** Clinical data and follow-up immunologic investigations of 5 children with cblC deficiency

Gender	P1	P2	P3	P4	P5
	F	F	F	F	M
Age	1y3m	1y8m	3 m	5y7m	4y1m
*MMACHC* variants	c.80 A > G/c.452 A > G	c.609G > A/c.80 A > G	c.609G > A/c.658_660delAAG	c.609G > A/c.80 A > G	c.609G > A/c.80 A > G
Total T lymphocytes (%)	57.6 (53.37–71.91)	79.8 (53.37–71.91)	84.8 (74.15–82.16)	70.5 (53.37–71.91)	65.5 (55.0–82.0)
CD3 + CD4+ lymphocytes (%)	41 (51.37–58.57)	51.8 (26.19–45.48)	48.1 (51.37–58.57)	43.7 (26.19–45.48)	30.0 (33–44)
CD3 + CD8+ lymphocytes (%)	14 (16.29–29.88)	29.3 (16.29–29.88)	31.6 (17.27–27.18)	24.5 (16.29–29.88)	25.4 (8.0–31.0)
B lymphocytes (%)	40 (13.93–30.49)	16.4 (13.93–30.49)	8.4 (7.49–17.71)	23.3 (13.93–30.49)	25.7 (11.0–45.0)
Natural killer cells (%)	1.4 (6.53–22.24)	3.6 (6.53–22.24)	4.9 (6.92–14.19)	5.0 (6.53–22.24)	5.8 (7.0–40.0)
Total T lymphocytes (cells/ul)	1378 (1775–3953)	2507 (1775–3953)	1401 (1744–3226)	2300 (2980–5950)	2222 (1794–4247)
B lymphocytes (cells/ul)	957 (537–1464)	515 (537–1464)	245 (292–858)	536 (537–1464)	722 (461–1456)
Natural killer cells (cells/ul)	33 (241–978)	113 (241–978)	143 (266–602)	115 (241–978)	458 (270–1053)
CD4/CD8	2.93 (1.05–2.53)	1.77 (1.05–2.53)	1.52 (1.97–3.32)	1.78 (1.05–2.53)	1.18 (1.1-2.0)
IgA (g/L)	0.65 (0.3–1.2)	< 0.0667 (0.3–1.2)	< 0.0667 (0.07–0.5)	0.672 (0.4–1.8)	1.47 (0.4–1.8)
IgG (g/L)	3.71 (3.5–10)	3.41 (3.5–10)	3.83 (2.5–7.5)	4.34 (5–13)	3.07 (5–13)
IgM (g/L)	0.901 (0.4–1.4)	0.487 (0.4–1.4)	0.091 (0.1–0.7)	1.38 (0.4–1.8)	0.799 (0.4–1.8)
IgE (IU/ml)	67 (< 60)	33.5 (< 60)	< 5.00 (< 15)	20.5 (< 60)	66.8 (< 60)
WBCs (×10^9^/L)	3.66 (4.0–10.0)	3.89 (4.0–10.0)	2.7 (4.0–10.0)	4.19 (4.0–10.0)	4.95 (4.0–10.0)
ANC (×10^9^/L)	1.97 (0.72–4.6)	1.43 (0.72–4.6)	0.65 (0.72–4.6)	2.89 (0.72–4.6)	1.47 (0.72–4.6)
ALC (×10^9^/L)	1.04 (1.48–7.8)	2.0 (1.48–7.8)	1.46 (1.48–7.8)	0.56 (1.48–7.8)	3.2 (1.48–7.8)
Follow-up immunologic data					
Follow-up age	3y9m	3y6m	1y2m	6y5m	4y7m
Total T lymphocytes (%)	51 (53.88–72.87)	63.7 (53.37–71.91)	45.9 (53.88–72.87)	NA	69.8 (53.37–71.91)
CD3 + CD4+ lymphocytes (%)	28.9 (24.08–42.52)	41.7 (26.19–45.48)	23.6 (24.08–42.52)	NA	41.8 (26.19–45.48)
CD3 + CD8+ lymphocytes (%)	15.3 (19.00-32.51)	13.4 (16.29–29.88)	18 (19.00-32.51)	NA	22.2 (16.29–29.88)
B lymphocytes (%)	39 (13.23–26.39)	29.7 (13.93–30.49)	37.9 (13.23–26.39)	NA	15.7 (13.93–30.49)
Natural killer cells (%)	5.9 (7.21–20.90)	2.3 (6.53–22.24)	12.4 (7.21–20.90)	NA	11.2 (6.53–22.24)
Total T lymphocytes (cells/ul)	3855 (1794–4247)	1949 (1775–3953)	3769 (1794–4247)	NA	4061 (1775–3953)
B lymphocytes (cells/ul)	2734 (461–1456)	969 (537–1464)	2969 (461–1456)	NA	908 (537–1464)
Natural killer cells (cells/ul)	414 (270–1053)	75 (241–978)	972 (270–1053)	NA	648 (241–978)
CD4/CD8	1.89 (0.90–2.13)	3.11 (1.05–2.53)	1.31 (0.90–2.13)	NA	1.88 (1.05–2.53)
IgA (g/L)	0.49 (0.4–1.8)	0.2 (0.4–1.8)	0.42 (0.19–1.75)	0.7 (0.4–1.8)	1.33 (0.4–1.8)
IgG (g/L)	5.05 (5–13)	11.3 (5–13)	6.15 (3.5–10)	8.21 (5–13)	11.7 (5–13)
IgM (g/L)	0.987 (0.4–1.8)	1.59 (0.4–1.8)	1.29 (0.4–1.4)	1.24 (0.4–1.8)	1.53 (0.4–1.8)
IgE (IU/ml)	168 (< 60)	30.1 (< 60)	38.8 (< 60)	56 (< 60)	126 (< 60)

*Note*: *WBCs *White blood cells, *ANC *Absolute neutrophil count, *ALC *Absolute lymphocyte count, *Ig *Immunoglobulin, *NA *Not available

In cblC deficiency, the accumulation of toxic metabolites such as methylmalonic acid and propionylcarnitine may disrupts mitochondrial energy production in immune cells, resulting in impaired immune maturation and function. This metabolic disturbance may hinder T cell proliferation and differentiation, compromise B cell antibody production, and reduce phagocytic function of innate immune cells, including neutrophils and monocytes. As an inborn error of intracellular cobalamin metabolism, the secondary accumulation of toxic organic acids in cblC deficiency induces mitochondrial dysfunction, which subsequently impairs the metabolic reprogramming essential for immune cell activation, particularly T lymphocytes [4]. These disruptions contribute to chronic systemic inflammation, immune dysregulation, and diminished capacity to recover from immune stress.

All patients received metabolic therapy together with anti-infective treatment during the infection. No cases of serious opportunistic infection were identified in our case series. Notably, none of the patients received immunoglobulin replacement therapy. In the available paired follow-up assessments, serum IgG concentrations were within the corresponding age-specific reference ranges in all five patients, and improvement in some T- and B-lymphocyte counts was also observed (Table 1). However, these changes occurred concurrently with clinical recovery from infection and stabilization of the metabolic condition. These observations suggest that at least some of the immunologic abnormalities in cblC deficiency may be reversible or may fluctuate with the patient’s clinical and metabolic status. Larger prospective studies with standardized metabolic and immunologic assessments are required to clarify this relationship.

Given this underrecognized immune phenotype, we emphasize the importance of considering inborn errors of metabolism in pediatric patients presenting with immunodeficiency-like features. When evaluating pediatric patients with suspected CVID or combined immunodeficiency, clinicians should maintain a high index of suspicion for an underlying metabolic phenocopy if atypical multisystem features are present. These include unexplained macrocytic anemia, neurodevelopmental delay, metabolic acidosis, hemolytic uremic syndrome, pulmonary hypertension, diffuse lung disease, or ocular involvement. Crucially, because *MMACHC* may not be included in standard targeted IEI gene panels, relying solely on these targeted assays may delay diagnosis. Therefore, when an underlying metabolic phenocopy is suspected, or when targeted IEI panels yield negative results in a highly symptomatic patient, *MMACHC* coverage should be verified or broader testing such as whole exome or genome sequencing should be considered.

## Data Availability

The data that support the findings of this study are available from the corresponding author upon reasonable request, subject to applicable ethical and privacy restrictions.
